# Up regulation of long non-coding RNAs BACE1 and down regulation of LINC-PINT are associated with CRC clinicopathological characteristics

**DOI:** 10.1007/s11033-022-07707-4

**Published:** 2022-09-10

**Authors:** Sara Bakhtiari-Nezhad, Leili Rejali, Mahrooyeh Hadizadeh, Mohammad Yaghob Taleghani, Hamid Asadzadeh Aghdaei, Chris Young, Binazir Khanabadi, Ehsan Nazemalhosseini-Mojarad, Maziar Ashrafian Bonab

**Affiliations:** 1 Basic and Molecular Epidemiology of Gastrointestinal Disorders Research Centre, Research Institute for Gastroenterology and Liver Diseases, Shaheed Beheshti University of Medical Sciences, Tehran, Iran; 2 Faculty of Health and Wellbeing, Canterbury Christ Church University, North Holmes Road, Canterbury, CT1 1QU UK; 3 Leicester School of Allied Health Sciences, Faculty of Health and Life Sciences, De Montfort University, Leicester, LE1 9BH UK; 4 Gastroenterology and Liver Diseases Research Centre, Research Institute for Gastroenterology and Liver Diseases, Shaheed Beheshti University of Medical Sciences, Tehran, Iran; 5 Kent and Medway Medical School, Pears Building, Park Wood Road, Canterbury, Kent CT2 7FS UK

**Keywords:** Colorectal cancer (CRC), Long non-coding RNAs (LncRNAs), LINC-PINT, LNC-BACE1

## Abstract

**Background:**

Long non-coding RNAs (LncRNAs) are known to have regulatory consequences for aberrant gene expression in cancers. The aim of this study was to evaluate the expression levels of long non-encoding RNAs, BACE1 (β-secretase1) and LINC-PINT (Long Intergenic Non-Protein Coding RNA, P53 Induced Transcript), in colorectal cancer (CRC) with clinicopathological parameters.

**Methods and results:**

Bioinformatics analysis defining effectual signalling pathways Wnt. A total of 130 tissue samples (50 fresh CRC tissues with parallel adjacent normal tissues (ADJ) accompanied with 30 normal healthy control tissue samples) were collected from the Iranian population. mRNA expression analysis was performed via Real Time Q-PCR. Statistical analysis for comparing CRC expression levels with ADJ and normal healthy tissues were carried out using Kruskal–Wallis tests. The Receiver Operating Characteristic (ROC) curve was plotted for each LNC, separately. We discovered that PINT and BACE1 expression levels were decreased and increased respectively in CRC tumour samples compared with ADJ normal and healthy tissues. Clinicopathological parameter assessment revealed a significant relationship between PINT expression, tumour location, staging and distant metastasis (p < 0.009, p < 0.014, p < 0.008, respectively). Also, BACE1 over expression was significantly associated with tumour site (p < 0.009), metastasis (p < 0.017) and histological differentiation (p < 0.028) and staging (p < 0.017). Furthermore, ROC curve plotting showed LINC-PINT LNC-BACE1 may distinguish between early and late-stage of CRC, highlighting the value of both BACE1 and PINT as CRC progression biomarkers.

**Conclusion:**

We investigated two LNCRNAs (PINT and BACE1) as potential CRC prognostic biomarkers, which are imperative for early and effective medical intervention in CRC. Expression levels of PINT and BACE1 in CRC tissue samples may serve to identify metastasis earlier, increasing patient survival rates and expediating clinical treatment options.

**Supplementary Information:**

The online version contains supplementary material available at 10.1007/s11033-022-07707-4.

## Introduction

Colorectal cancer (CRC) is one of the most prevalent and lethal cancers [1–3] and the second leading cause of death in both males and female cancer patients, including 11% of cancer dependence patients [4, 5]. CRCs are formed by the growth of colon, rectal, and appendix cells [6, 7]. Patients with lack of lymph node metastasis can undergo simple surgery, including removing the tumour along with proximal tumoural tissues, which has been shown to increase survival rates up to 80% [7, 8], but if the malignancy had spread to the lymph nodes, the rate of survival drops to 32–42% [8]. This illustrates the importance of predicting lymph node involvement prior to metastasis in CRC patients to improve survival rates and facilitate appropriate treatment options.

In recent years, many biomarkers have been proposed, including for the detection of mutations in relevant genetic elements, evaluating genetic regulators, and examining microsatellite instability. For a comprehensive review, see Veltman and Brunner [9–13].

LncRNAs have a length of more than 200 nucleotides and have numerous biological roles, such as regulating the expression of genes, modifying genes through epigenetics and regulatory roles during and after transcription [14–18]. The expression of LncRNAs linked with colorectal cancer has been documented previously, the p53 gene is an example of a well-known inhibitory gene, which played a vital role in genomic stability and tumour suppression, mainly by inducing apoptosis, cessation of the cell cycle, aging, and apoptosis inhibition [19, 20]. LINC-PINT induces apoptosis and increases survival by regulating the expression of the p53 gene. Increasing the expression of LINC-PINT has a positive self-regulatory effect on P53, inducing apoptosis in tumour cells and improving survival rates in cancer patients [21]. Increased levels of the glycosyltransferase enzyme ST6GAL1 are often associated with elevated tumour grade and metastasis, and patient prognosis. BACE1, through degradation, can reduce ST6GAL1 enzyme in cancer cells, rendering its inhibitory role in tumour metastasis diminished [22, 23]. Therefore, identifying the molecular mechanisms of BACE1 expression can help in detecting malignancy in the large intestine [24, 25]. Increasing BACE1 levels have previously been shown to reduce ST6GAL1 enzyme expression and metastasis in colon cancer cases [26].

The aim of this study was to evaluate the expression level of LINC-PINT and BACE1 in terms of clinicopathological parameter association, specifically lymph node involvement in CRC affected patients, which has not previously been addressed in the literature.

## Materials and methods

### Bioinformatics analysis

Based on high-throughput data bases like: GeneCards, NONCODE, LncRNA Disease and The Cancer Gene Atlas databases, selected LncRNAs, BACE1 & PINT matched with the data obtained from the signalling pathways database.

### Patient’s selection

In this study, 50 fresh CRC tissue samples were selected from patients who had been referred to Taleghani Hospital (Tehran, Iran) for CRC free screening program, along with adjacent normal tissue samples, with following criteria: (1) availability of tissue samples and medical history, (2) availability of follow-up data, and (3) absence of severe perioperative complications or (4) no records of radiotherapy and chemotherapy previously received. Candidates missed their follow‐up were omitted from the survey. 30 normal (control) tissue samples were chosen with respect to their normal report of colonoscopy without any inflammation, any polyp or tumour and without recorded history notification of personal or familial cancer.

Patients were randomly chosen and 68% were categorized in early CRC TNM stages (I&II) and 32% were diagnosed at late stages of disease (III&IV). Personal written consent was received from all enrolled cases according to Helsinki Declaration guidelines [27]. The study was approved by the Institutional Research Ethics Committee of Taleghani Hospital (Ethical approval number: IR.SBMU.RIGLD.REC.1396.180). Tumoural and adjacent normal tissue distinction and confirmation was cooperated by two expert pathologists. Data analysis was carried out using SPSS and GraphPad Prism 8.

### RNA extraction and analysis

For RNA extraction and purification, resected tissues were defrosted from − 70 °C and RNA extractions were performed using of RNeasy mini kit (Qiagen). Quality and quantity of purified RNA was measured by nanodrop. After optical density assessment, cDNA was synthesized using PrimeScript cDNA synthesis kit (Takara, Da-lian, China). Real Time PCRs were performed using SYBR Green Real-Time PCR Master Mix (Takara, Da-lian, China). All reactions were run using a Rotor-Gene light cycler (Qiagen, Hilden, Germany) as previously described [28]. Specific designed primers for LncRNAs detection are listed in Supplementary Table 1S. Thermal cycling conditions were as follows: 30 s at 95 °C, 95 °C for 5 s, 58 °C for 34 s, and a primer extension 60 °C for 34 s and 15 s at 95 °C, for 40 cycles. To ensure cDNA synthesis quality and gene expression comparison, 18S rRNA was used as an internal control [29].

### Fold change calculations and statistical analysis

LncRNA expression assessments between tumoural, adjacent normal and normal tissue groups were performed and the fold change or RQ validation was determined by using (2^−ΔΔCT^) method and by use of nonparametric Kruskal–Wallis test and Mann–Whitney where ever needed. The log rank test was performed for determining statistical significance. A significant relationship between the target and control groups was observed by RQ values. RQ < 0.5 was interpreted as a decrease in the expression level and RQ value > 2 assign as an increase in gene expression [30]. Receiver Operating Characteristic (ROC) curves for early stages and late stages were drawn, and mean RQs calculated.

## Results

### Lnc selection by use of bioinformatics

**Fig. 1:**
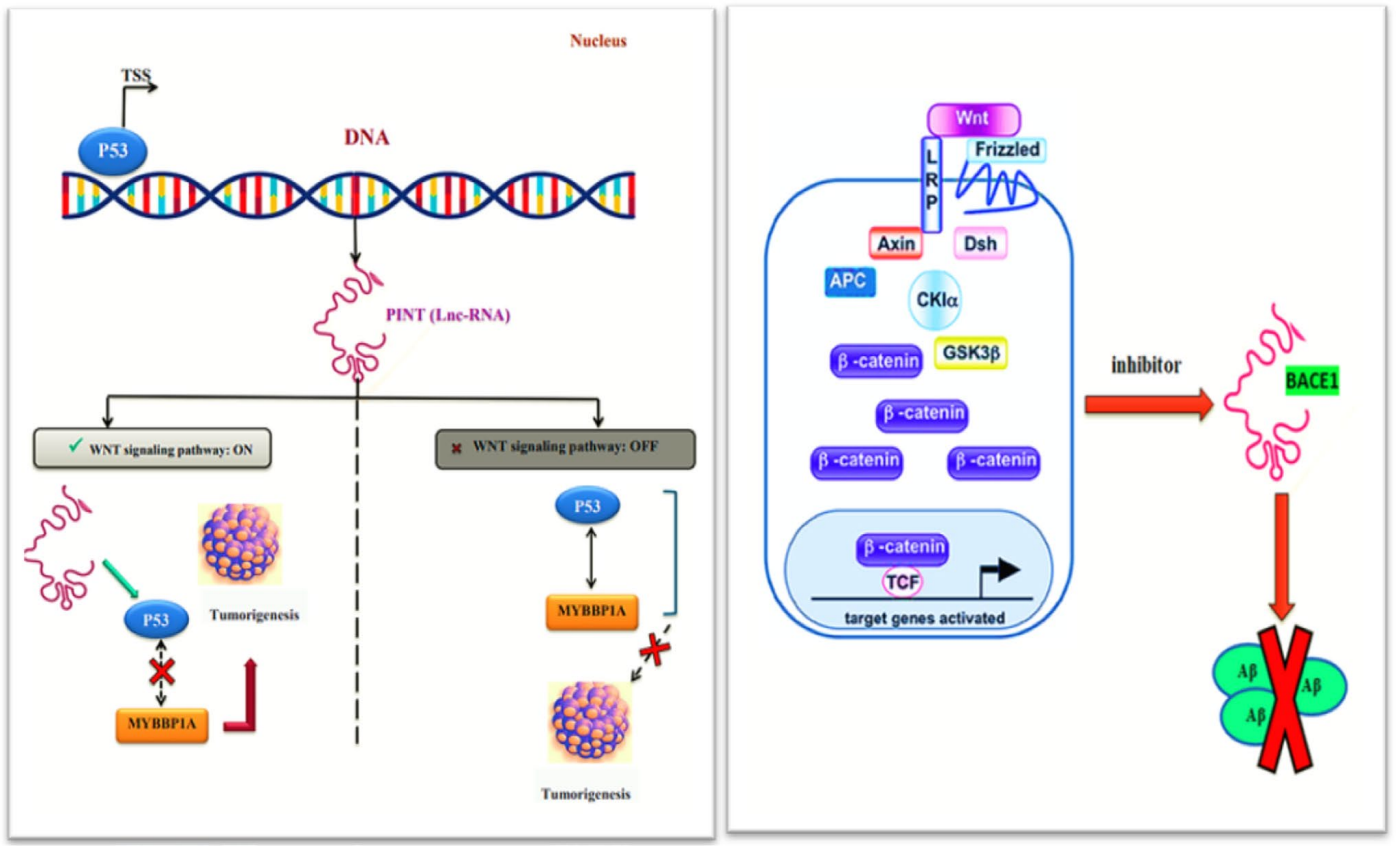
LINC-PINT and LNC-BACE1 roles in carcinogenesis signalling pathways. **A** LINC-PINT is significantly play role in Wnt/β-Catenin by inhibiting the p53-MYBBP1A complex formation. **B** LNC-BACE1 is defined in Wnt/β-Catenin signalling pathway via reducing Aβ42

Signalling pathway data base interrogation showed that LINC-PINT plays a role in Wnt/β-Catenin signalling. LINC- PINT, regulated by p53, inhibited the basal p53 levels and facilitated tumorigenicity in colorectal cancer via associating with MYBBP1A and inhibiting the p53-MYBBP1A complex formation. Furthermore, LNC-BACE1 potentially reduces expression in the JAK2/STAT3 pathway, eventually leading to metastasis in CRC. In this regard, activation of Wnt/β-catenin signalling reduces Aβ42 production and aggregation, Wnt inhibition induces the opposite effect on APP processing and Aβ42 production/aggregation in a cellular model (Fig. 1).

### Sample collection parameters

Fifty confirmed CRC diagnosed patients were enrolled according to inclusion and exclusion criteria previously described. 56% of selected samples were male (28) and 44% were female (22). Patients with rectosigmoid tumour grab the highest frequency versus patients with cecum tumour who demonstrated the lowest prevalence of tumour location. Near 40% of enrolled patient’s samples were moderately differentiated and around 30% illustrated poor dysplasia. CRC patient’s samples were categorized in four groups according to determinate pathologic stage of disease. Patients detected in the survey were mostly in early stage of disease (I&II) and close to 30% were in advance stage (III&IV). Based on metastasis classification, samples were divided to two groups of present or absent of metastasis in advance stages. The outcome indicated only 10% of patients with distant metastastatic cells (5 cases) and 90% were distinguished without metastasis (45 cases). Data were categorized in Supplementary Table 2S.

### LINC-PINT acts as tumour suppressor but LNC-BACE1 shows an oncogene role in CRC

**Fig. 2:**
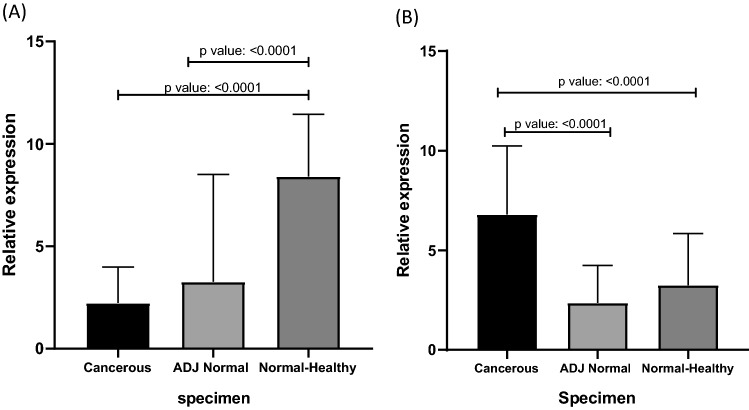
The comparison between cancerous, ADJ normal and normal healthy specimen was performed. The Kruskal–Wallis test is performed in **A** LNC-PINT (p value: < 0.0001) and **B** LNC-BACE1 (p value < 0.0001). Between each two categories Mann–Whitney test was established. *p value under 0.05 considered significant

**Fig. 3:**
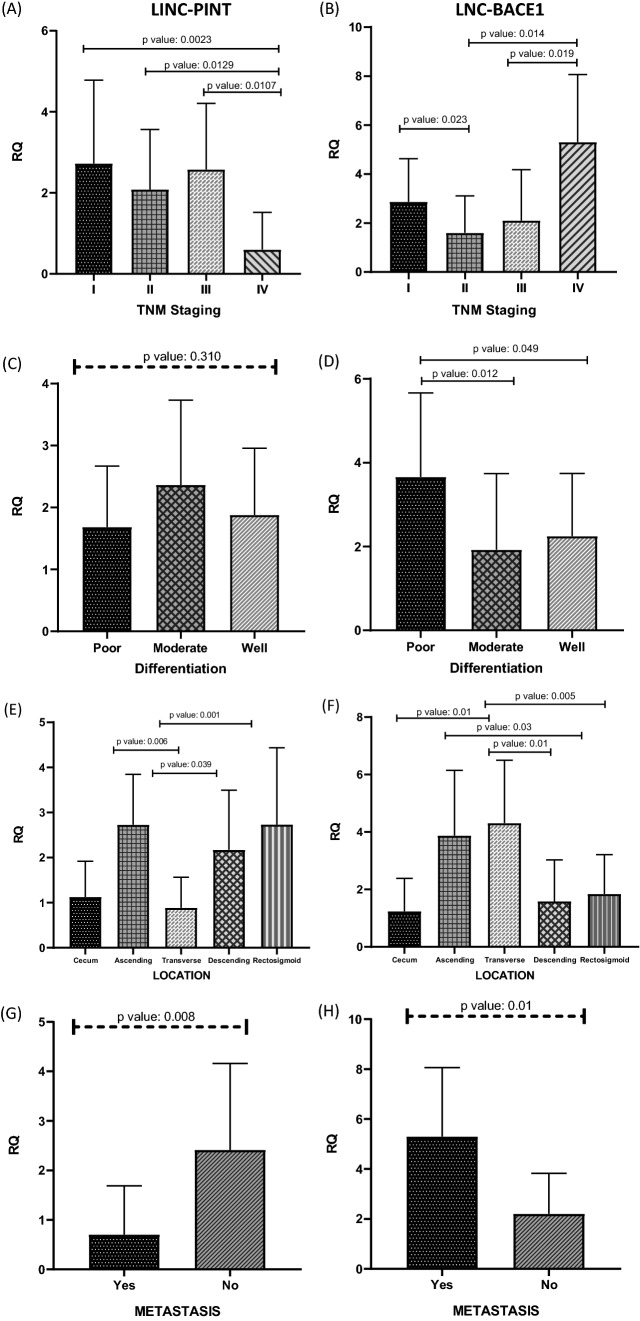
Clinicopathological characteristic comparison in Left column LINC-PINT and Right column LNC-BACE1. **A, B** TNM staging of CRC was compared between four stages of disease in PINT and BACE1. **C, D** Histologic differentiation of both LNCs (PINT & BACE1) was statistically analysed by kruskal–wallis test. **E, F** The location of resected tumours was separately analysed and compared by RQ (Relative Quantification) of each LNC (PINT & BACE1). **G, H** Distant metastasis was considered as a main parameter in our survey, PINT and BACE1 both display significant p value in Mann–Whitney statistical analysis. *p value under 0.05 considered significant

Expression levels of LINC-PINT were downregulated in CRC tumour tissues compared with adjacent normal tissues, where 56% of cancerous patients demonstrated a decrease and 44% of patients had an increase in the mRNA level of LINC-PINT. mRNA expression evaluation of LINC-PINT in cancerous, ADJ Normal and normal-healthy tissues were statistically analysed and the significant p value between cancerous and normal healthy specimens and fantastically ADJ normal versus normal-healthy was distinguished. (p value: < 0.0001) (Fig. 2A). In terms of the association determination of LINC-PINT with clinicopathological parameters, down regulations in expression levels of LINC-PINT were significantly associated with CRC stage (p value: 0.01), tumour location (p value: 0.02) and distant metastasis (p value: 0.008) (Fig. 3A, E, G). The PINT reduction trend is significantly related to histopathological stage advancement. The tumours located at the transverse of colon were significantly shows down regulation in PINT mRNA expression compared with ascending, descending and rectosigmoid sections (p value: 0.009). There is also significant down regulation of PINT in transverse section of colon in comparison with other parts of colon. In ascending (p value: 0.006), descending (p value: 0.039) and rectosigmoid (p value: 0.001) upregulation was perceived. Patients with distant metastasis demonstrated the significant reduction of PINT. (p value: 0.008) (Table 1).

**Table 1:** LINC-PINT & LNC-BACE1 relationship with clinicopathological parameters

	Up regulate PINT (%)	Down regulate PINT (%)	p value PINT	Down regulate BACE (%)	Up regulate BACE (%)	p value BACE
Patients	24(48)	26(52)		21(42)	29(58)	
Age						
< 55	11 (22)	12 (22)		9 (18)	14 (28)	
> 55	13 (26)	14 (30)	0.319	12 (24)	15 (30)	0.271
Gender						
Female	11 (22)	11(22)	0.233	13 (26)	9 (18)	0.147
Male	13 (26)	15 (30)		11 (22)	17 (34)	
Location of tumour						
Cecum	–	4 (8)		1 (2)	4 (8)	
Ascending	4 (4)	3 (10)		4 (8)	2 (4)	
Transverse colon	10 (12)	5 (10)	0.01*	3 (6)	5 (10)	< 0.001*
Descending	4 (4)	3 (10)		6 (12)	1 (2)	
Rectosigmoid	6 (6)	11 (42)		9 (18)	15 (30)	
Differentiation						
Poor	8 (16)	6 (12)		6 (12)	7 (14)	
Moderate	6 (12)	15 (30)	0.02*	9 (18)	12 (24)	0.483
Well	10 (22)	5 (10)		9 (18)	7 (14)	
TNM stage						
I	6 (6)	2 (4)		5 (10)	–	
II	7 (10)	6 (14)		9 (18)	3 (6)	
III	8 (16)	12 (30)	< 0.001*	17 (34)	6 (12)	0.27
IV	3 (6)	6 (14)		8 (16)	2 (4)	
Metastasis						
Yes	6 (8)	1 (2)		5 (10)	–	
No	18 (36)	25(54)	< 0.001*	12 (24)	33 (66)	< 0.001*

*Significant p value (< 0.05)

In another hand, BACE1 with 65% over-expression in cancerous cases versus normal samples were designated as an oncogene in LNC categories. In comparison between cancerous specimen and ADJ normal as well as cancerous and normal-healthy samples significant p value (< 0.0001) were recognized (Fig. 2B). Over expression of BACE1 developed significant relations with TNM staging of disease (p value: 0.01), tumour histological differentiation (p value: 0.02), growing tumour location (p value: 0.009) and far apart metastasis (p value: 0.01) (Fig. 3B, D, F, H). BACE1 expression elevation in last stage of disease was significantly associated with stage II and early advance stage III. In another hand over expression of BACE1 in poorly differentiated tumours were significantly associated with moderate or well differentiated samples. It is interesting that BACE1 over expression was significantly observed in transverse resected tumours where it could be compared with PINT with down regulation. Transverse tumours are obviously showed the trend of expression. Again, in BACE1 like PINT, transverse specimen over expression demonstrated significant p value compared with descending (p value: 0.01) and rectosigmoid (p value: 0.005) tumours but no obvious changes were seen with ascending samples. Furthermore, cecum samples with down regulation are significantly lowered versus transverse over expression (p value: 0.01). As well as PINT, over expression of BACE1 is significantly associated with distant metastasis in CRC tumours (Table 1).

**Fig. 4:**
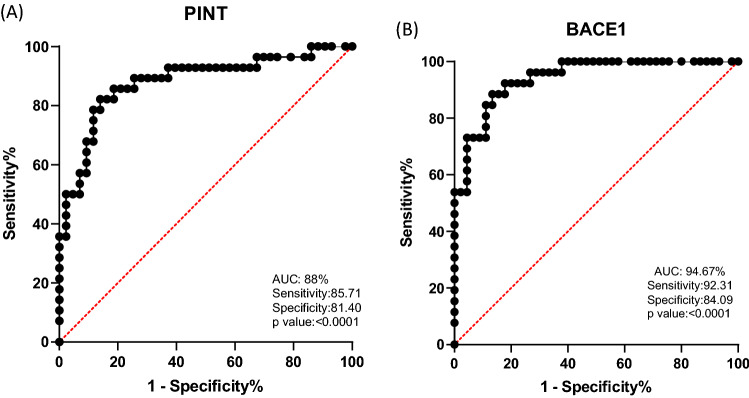
The ROC curve of PINT and BACE1in colorectal cancer cohorts plotted between early and late stage of CRC disease. **A** The ROC graph was plotted for LINC-PINT, AUC was estimated 88.04 with e sensitivity and specificity (85.71% and 81.40%, respectively) and **B** the BACE1 AUC (Area Under Curve) was 94.67, with sensitivity (92.31%) and specificity (84.09%). Both p values were designated significant < 0.0001. *p value under 0.05 considered significant

ROC curves were employed to assess the diagnostic and prognostic potential of BACE1 and LINC-PINT as biomarkers (Fig. 4). LINC-PINT AUC was 88.04 with sensitivity and specificity (85.71% and 81.40%, respectively). Our data showed BACE1 area under curve (AUC) was 94.67, demonstrating a high possibility of detection between late and early stages, also high sensitivity (92.31%) and specificity (84.09%) were distinguished.

## Discussion

Significant recent efforts have been made to identify the mechanisms underlying the elevation in large intestine incidences of mortality and morbidity, using changes in gene expression, active mutations, evaluation of non-encoding RNAs and the relationship with lymph node metastasis, in order to earlier diagnose the stage of disease and commence more immediate treatment. Moreover, novel findings around LncRNA participation in Wnt/β-Catenin signalling pathways provide new insight into the relationship between lymph node metastasis and expression level of BACE1 and LINC-PINT. This will better inform clinicians aiming to incorporate molecular data with pathology to increase treatment options and patient outcomes.

In the present study, more than 55% of patients displayed reduced LINC-PINT expression, this downregulation was significantly related to pathological information including tumour location, tumour stage and differentiation. Metastasis, which is a primary factor in cancer progression, was significantly associated with BACE1 and LINC-PINT expression. According to previous studies, LINC-PINT is a transcript inducer of oncogene P53, during suppression of PINT, oncogene P53 is impressive on the progression of cancer and tumour development [14, 19, 31]. Another study reported that LINC-PINT was downregulated in plasma and associated with tumour recurrence in patients with pancreatic cancer [30, 32]. In the present study, LINC-PINT mRNA expression level was significantly associated with variables such as tumour differentiation and tumour stage. LINC-PINT, which displays low expression levels in tumours, operates as a suppressor of cancer progression and an inverse relationship between the expression of LINC-PINT and the aggressiveness of the tumours exists [33]. Studies on LINC-PINT have shown that, by increasing the expression of LNC RNA PINT with a positive self-regulating effect on P53, tumour cell invasion was prevented [31, 34]. This agrees with our own findings where a decrease in LNC-PINT expression in cancer cells, and therefore metastasis expansion is expected. In the present study, a decrease in the level of LNC RNA PINT expression was observed in the rectosigmoid position of affected patients. Therefore, to some extent, it is possible to investigate the specificity of LNC PINT expression in colorectal cancer.

Recent studies have shown that increases in the level of STGAL1 enzyme are linked to reduced tumour metastasis. Increasing the level of BACE1 reduces STGAL1 enzyme expression, thus eliminating the important role of this enzyme in reducing tumour metastasis [9]. In this study, there was a significant correlation between the level of expression of BACE1 and lymph node involvement in patients with colorectal cancer. Therefore, the importance of examining the level of expression of these two LncRNAs in patients with lymph node involvement has been investigated. In our study, BACE1 expression levels were reduced in tumoural tissues with metastasis, while in another study on cancerous liver patients, BACE levels were increased. Therefore, it is likely that the expression level of each LncRNA varies in different cancers/tissues.

We found the expression of BACE1 was significantly higher in the tumoural metastasis tissues and adjacent normal tissues than control tissues. However, a previous report showed that LncRNA BC039913 expression was associated with a reduction in cancer cells, but no significant association was found between tumour formation and metastasis. Also, there was no significant relationship between age and sex indicators with LncRNA BC039913 expression, which is consistent with our study [35, 36]. In 2019, Hong et al. also reported that PTCS3 interacts with LINCPINT to prevent tumour growth in gastric cancer, and the expression level of PTCS3 in tumour tissue was significantly correlated with the expression level of LNC PINT in adjacent normal tissue [37]. Although expression levels in CRC and normal adjacent tissues were not significantly associated with gender or age of samples selected, similarly to other reported data, CRC affected men more than women in these obtained statistics.

In the present study, the potential of LINC-PINT and BACE1 for use as diagnostic biomarkers in CRC was evaluated. ROC curve analysis indicated sensitivity and specificity in LINC-PINT and BACE1 for colorectal cancer diagnosis. Despite colonoscopy being the general and popular choice among clinicians for CRC screening, non-invasive molecular biomarkers could also be efficient alongside invasive colonoscopy procedures. Here, we have demonstrated the potential for PINT and BACE1-AS as molecular biomarkers, but more data is needed to confirm the molecular mechanisms at play here.

## Conclusions

LncRNAs play an important role in cell proliferation and regulation, including growth, differentiation. In this study, we have shown a new link between lymph node involvement in patients with colorectal cancer and the levels of LINC-PINT and BACE1 mRNA expression in CRC tumour samples. These novel potential biomarkers could be used to diagnose CRC patients earlier, for more effective and less invasive treatment.

## Supplementary Information

Below is the link to the electronic supplementary material.

Supplementary file1 (DOCX 16 KB)
