# Supplementary material for: Up regulation of long non-coding RNAs BACE1 and down regulation of LINC-PINT are associated with CRC clinicopathological characteristics
Source: Mol Biol Rep. 2022 Sep 10;49(11):10259–67. doi: 10.1007/s11033-022-07707-4 (PMC9618545; doi:10.1007/s11033-022-07707-4)
Supplement: Supplementary file 1 — Supplementary file1 (DOCX 16 KB) [file 11033_2022_7707_MOESM1_ESM.docx]

**SUPPLEMENTARY:**

**Table 1S**: Primer sequences

| **Gene ID** | **Primer** | **Sequence** |
| --- | --- | --- |
| 23621 | BACE1 | 5′- GTCCCAAGACGACTGTTACAA-3′  5′- CCCGATCAAAGACAACGTAGAA-3′ |
| 378805 | PINT | 5′- AAAGCCGTGGTGATGGTAAT -3′  5′- GATTTGTCCCTCTGCGAGTT -3′ |
| 100008588 | 18S rRNA | 5′-CACGGACAGGATTGACAGATT-3′  5′-GCCAGAGTCTCGTTCGTTATC-3′ |

**Table 2S:** Characteristics of the enrolled CRC patients.

| **Characteristics** | **No. of Cases** | **%** |
| --- | --- | --- |
| **Gender:** |  |  |
| Male | 28 | (56) |
| Female | 22 | (44) |
| **Age (years):** |  |  |
| ≤55 years | 23 | (46) |
| >55 years | 27 | (54) |
| **Histological grade:** |  |  |
| Grade I (well differentiated) | 17 | (34) |
| Grade II (moderately differentiated) | 18 | (36) |
| Grade III (poorly differentiated) | 15 | (30) |
| **Pathological stage:** |  |  |
| Stage I | 17 | (34) |
| Stage II | 17 | (34) |
| Stage III | 11 | (22) |
| Stage VI | 5 | (10) |
| **Lymph node involvement:** |  |  |
| No | 45 | (90) |
| Yes | 5 | (10) |
| **Localization:** |  |  |
| Cecum | 2 | (4) |
| Ascending | 5 | (10) |
| Transverse colon | 6 | (12) |
| Descending | 5 | (10) |
| Rectosigmoid | 32 | (64) |
